# Shot Boundary Detection with 3D Depthwise Convolutions and Visual Attention

**DOI:** 10.3390/s23167022

**Published:** 2023-08-08

**Authors:** Miguel Jose Esteve Brotons, Francisco Javier Lucendo, Rodriguez-Juan Javier, Jose Garcia-Rodriguez

**Affiliations:** 1 Telefónica I+D, 28050 Madrid, Spain; miguel.estevebrotons@telefonica.com (M.J.E.B.); javier.lucendodegregorio@telefonica.com (F.J.L.); 2 Computers Technology Department, University of Alicante, 03080 Alicante, Spain; jrodriguez@dtic.ua.es

**Keywords:** shot boundary detection, 3D convolution, depthwise convolution, visual attention

## Abstract

Shot boundary detection is the process of identifying and locating the boundaries between individual shots in a video sequence. A shot is a continuous sequence of frames that are captured by a single camera, without any cuts or edits. Recent investigations have shown the effectiveness of the use of 3D convolutional networks to solve this task due to its high capacity to extract spatiotemporal features of the video and determine in which frame a transition or shot change occurs. When this task is used as part of a scene segmentation use case with the aim of improving the experience of viewing content from streaming platforms, the speed of segmentation is very important for live and near-live use cases such as start-over. The problem with models based on 3D convolutions is the large number of parameters that they entail. Standard 3D convolutions impose much higher CPU and memory requirements than do the same 2D operations. In this paper, we rely on depthwise separable convolutions to address the problem but with a scheme that significantly reduces the number of parameters. To compensate for the slight loss of performance, we analyze and propose the use of visual self-attention as a mechanism of improvement.

## 1. Introduction

Shot boundary detection is an important task in video processing and computer vision, where the goal is to automatically identify the boundaries between consecutive shots in a video sequence. One of the techniques used for shot boundary detection is 3D convolution. Three-dimensional convolution applies a filter kernel to a three-dimensional input volume, such as a video sequence. The kernel slides over the input volume in three dimensions, computing a dot product at each position. The result of this operation is a new three-dimensional volume, which can be used to detect shot boundaries. The input volume is typically composed of a stack of video frames, where each frame is represented as a two-dimensional image. In a simple way, a 3D convolution allows for extracting spatiotemporal features that are relevant for shot boundary detection, such as changes in color, texture, or motion. One common approach is to use a thresholding technique to separate regions of the volume that correspond to different shots. Other techniques, such as clustering, can also be used to segment the output volume and detect shot boundaries. The effectiveness of 3D convolution for shot boundary detection depends on how the convolutional layers are stacked, the type of convolution chosen, the number of filters utilized at each stage of the stacks, and how additional techniques help generalization such as batch normalization, pooling, and dropout are utilized.

Depthwise convolution is a type of convolution operation used in convolutional neural networks (CNNs). In contrast to traditional convolution, which convolves an input with a set of learnable filters across all input channels, depthwise convolution applies a separate filter to each input channel.The operation involves sliding a 3D kernel over each channel of the input, performing an element-wise multiplication between the kernel and the corresponding channel, and summing the resulting values to produce a single output value for each position in the feature map. This process is repeated for each channel in the input, resulting in a set of intermediate feature maps. Depthwise convolution can be followed by a pointwise convolution, which applies a 1 × 1 × 1 convolution to combine the intermediate feature maps into a new set of features. This combination is also known as a depthwise separable convolution, and it is commonly used in architectures that require efficient computation, such as mobile or embedded devices. The advantages of depthwise convolution include reduced computation and memory requirements, as well as increased model efficiency. By reducing the number of parameters in the network, depthwise convolution can improve training speed and reduce overfitting. Depthwise convolution has been used in various architectures, such as MobileNet [1] and Xception [2], to achieve high accuracy on a variety of computer vision tasks with reduced computational cost and memory usage.

Residual visual attention refers to a mechanism that enhances the ability of a convolutional neural network (CNN) to focus on salient features in an image, while also maintaining the representation of the original input. This mechanism combines the concept of residual connections, which enable the network to learn the difference between the input and the output, with visual attention, which allows the network to selectively focus on relevant features. In a residual visual attention block, the input is passed through a series of convolutional layers, followed by a residual connection that adds the input back to the output of the convolutional layers. A visual attention mechanism is then applied to the output, which weights the importance of different spatial and temporal regions of the feature map based on their relevance to the task at hand. The visual attention mechanism can be implemented in different ways, such as using a spatial transformer network, which applies an affine transformation to the feature map to focus on a specific region of interest, or using a self-attention module, which computes attention weights based on the similarity between different feature map locations. By combining residual connections with visual attention, residual visual attention blocks allow the network to selectively focus on salient features, while still preserving the representation of the original input. This can lead to improved performance on tasks such as image classification, object detection, and segmentation. Residual visual attention has been used in various deep learning architectures, such as ResNet-SE [3], SENet [3], and SKNet [4], to achieve state-of-the-art performance on a range of computer vision tasks.

Some novel aspects of our work include the following:

1. Modifying some state-of-the-art architectures such as Transnet [5] and Transnetv2 [6] to utilize depthwise convolutions and analyze the impact in performance based on the reduction of network parameters that depthwise implies.
2. The usage of a residual self-attention block with attention map computation and the verification of the performance of the models.
3. We provide comparative results, testing the performance of the models in three different sets of experiments. A first set with no depthwise convolutions, a second set with only depthwise convolutions, and a third set where we combine depthwise convolutions and residual attention blocks.

The remaining of paper is organized as follows: Section 2 reviews related works. In Section 3, we present our motivations and some high-level considerations. Section 4 describes the general approach to decrease the number of parameters in the current state-of-the-art 3D convolution-based models for shot boundary detection, meanwhile keeping performance metrics. Section 5 describes our experimentation to validate our proposals. We finish with our conclusions and further work in Section 6.

## 2. Related Work

**Depthwise separable convolutions.** Since the development of convolutional networks, many studies have been carried out aimed at reducing the number of parameters to optimize the computational load and improve the inference speed.

Flattened convolutional neural networks are presented in [7]. Those are designed for fast feedforward execution. It consists of consecutive sequences of one-dimensional filters across all directions in 3D space to obtain comparable performance as conventional convolutional networks. The flattened convolution pipelines provide around two times speed-up during the feedforward pass compared to the baseline model due to the significant reduction of learning parameters.

Factorized Networks [8] introduce a similar factorized convolution as well as the use of topological connections. Inspired by [9], the 3D convolution operation in a convolutional layer can be considered as performing spatial convolution in each channel and linear projection across channels simultaneously. By unraveling them and arranging the spatial convolutions sequentially, the proposed layer is composed of a low-cost single intra-channel convolution and a linear channel projection.

Some Inception versions already explored ways to scale up networks with the aim of utilizing the added computation as efficiently as possible by suitable factorized convolutions and aggressive regularization. The inceptionV3 network [10] replaces the original inception module by three variants: one where each 5 × 5 kernel is replaced by two 3 × 3, another where nxn convolutions are factorized by 1 × 1, 1 × n, and n × 1, and a third where the module is added additional depth with expanded filter banks, 1 × 3 and 3 × 1. The InceptionV4 and Inception-Resnet networks [11], in addition to adding new blocks called stem, located at the beginning of the network or making grid reductions of the feature maps, as well as using residual connections such as those introduced by Resnet [12], also replace some inception modules in InceptionV3 with new additional factorizations and mixed-depthwise convolutions.

The Xception network [2] demonstrated how to scale up depthwise separable filters to outperform Inception V3 [10]. By using depthwise separable convolutions, Xception reduces the number of parameters in the network and improves the accuracy by increasing the representational power of the network. Additionally, Xception uses residual connections to improve the flow of gradients through the network, which can improve the training stability.

The work in [13] focuses on action classification evaluating state-of-the-art architectures trained until the moment with small HMDB-51 and UCF-101 datasets but using a new Kinetics Human Action Video dataset. The papers analyze 2D ConvNets with LSTMs ([14,15]) and 2D two-stream networks ([16,17]) with different types of stream fusion and C3D [18]. They introduce a Two-Stream Inflated 3D ConvNet (I3D), showing how 3D ConvNets can benefit from ImageNet 2D ConvNet designs and from their learned parameters.

In [19], authors take several variants of I3D in [13] and replace 3D convolutions with spatial and temporal separable 3D convolutions, replacing filters of the form kt × k × k by 1 × k × k followed by kt × 1 × 1, where kt is the width of the filter in time. K is the height/width of the filter in space. The resulting model is named S3D, which stands for “separable 3D CNN”, showing that it is more computationally efficient and with better accuracy than the original I3D model.

Ref. [20] proposes an architecture to reduce reasonably many parameters by simulating 3 × 3 × 3 convolutions with 1 × 3 × 3 convolutional filters on the spatial domain (equivalent to 2D CNN) plus 3 × 1 × 1 convolutions to construct temporal connections on adjacent feature maps in time. This approach is referred as the pseudo-3D convolution and is proposed a new architecture named Pseudo-3D Residual Net (P3D ResNet) that achieves clear improvements on a Sports-1M video classification dataset against standard 3D CNN. Pseudo 3D CNN not only reduces the model size significantly, but also enables the pre-training of 2D CNN from image data, endowing Pseudo 3D CNN more power to leverage the knowledge of scenes and objects learned from images.

The difference between separable and pseudo-3D CNNs is that in the latter, up to three types are defined depending on how the spatial and temporal convolutions are combined. The case of the pseudo block type A in [20] vertically stacks the spatial and temporal filters and is therefore completely equivalent to that of a separable 3D CNN.

Similar to [19,20], in [21], the authors factorize a standard 3D convolution into separate convolutions on separate channels and a pointwise convolution on all the channels.

Based in [10], MobileNet [1] is a popular architecture for efficient image classification that uses depthwise separable convolutions. The architecture was specifically designed for mobile and embedded devices where computational resources are limited.

ShuffleNet in [22] is another architecture that uses depthwise convolutions for efficient image classification. The architecture introduces a channel shuffle operation that helps to increase the accuracy of the network while maintaining a low computational cost.

MobileNetV2 in [23] is an improved version of MobileNet that uses a combination of depthwise and regular convolutions to achieve high accuracy on image classification tasks. The architecture introduces a novel inverted residual block that helps to reduce the number of parameters in the network while maintaining accuracy.

SqueezeNet in [24] is an architecture that uses a combination of depthwise and regular convolutions to achieve high accuracy on image classification tasks with a low computational cost. The architecture introduces a novel fire module that combines a squeeze layer, which uses depthwise convolutions, with an expanded layer, which uses regular convolutions.

In [5], shot detection is conducted using Dilated Convolutions, with different dilation rates for the time dimension in order to augment the receptive field without increasing the number of network parameters. Dilated Convolutions outputs are concatenated in the channel dimension forming a Dilated DCNN layer. Then, multiple Dilated DCNN layers are stacked with spatial max pooling to form a Stacked DDCNN block. The network proposed then consists of multiple SDDCNN blocks, with the very next block operating on a smaller resolution but a greater channel dimension. The network is trained using 3000 videos in the TRECVID IACC3 [25] dataset with the automatic creation of transitions. Evaluation was conducted using 100 IACC3 videos different from the training set and with testing conducted using RAI [26].

A second version of the network provided in [5] is presented in [6], which improves by adding Convolution Kernel Factorization, Frame Similarities as Features, and Multiple Classification Heads.

**Self-attention modules.** Attention modules can model long-range dependencies and have been widely applied in many tasks. In particular, the work [27] is the first to propose the self-attention mechanism to draw global dependencies of inputs and apply it in machine translation. The work in [28] introduces a self-attention mechanism into the generator network of the GAN. This allows the generator to attend to different parts of the image while generating it, making it possible to generate higher quality and more diverse images. The generator network is augmented with a self-attention module that calculates the importance of each spatial location in the feature maps, based on its relationship with all other locations. The resulting attention map is then used to weight the feature maps, allowing the generator to focus on important regions of the image while generating it. The work in [29] integrates two types of attention mechanisms: spatial and channel attention. Each of the attention blocks calculates an attention map, which is used to weigh the feature maps along its respective dimension. In [30], a residual self-attention deep neural network is proposed to capture local, global, and spatial information of magnetic resonance images to improve diagnostic performance. In [31], a very similar self-attention block as in [30] is proposed for complex human motion video classification.

Different from previous works, we extend the self-attention mechanism in the task of shot boundary detection and carefully design several types of attention modules to capture rich contextual relationships for better feature representations with intra-class compactness. Comprehensive empirical results in Section 5 verify the effectiveness of our proposed method.

## 3. Motivation and High-Level Considerations

The most straightforward way of improving the performance of deep neural networks is by increasing their size; this includes both increasing the depth (the number of levels) and its width (the number of units at each level). This is an easy and safe way of training higher quality models, especially given the ability of a large amount of labeled training data. However, this solution comes with two major drawbacks. A bigger size typically means a larger number of parameters, which makes the enlarged network more prone to overfitting, especially if the number of labeled examples in the training set is limited. Second, uniformly increased network size is the dramatically increased use of computational resources. The fundamental way of solving both issues would be by ultimately moving from fully connected to sparsely connected architectures, even inside the convolutions.

Sparsely connected refers to a type of neural network architecture where not all neurons in a layer are connected to every neuron in the previous layer. In other words, some connections between neurons are omitted or removed, resulting in a sparse connectivity pattern. In contrast, a fully connected neural network architecture has every neuron in one layer connected to every neuron in the next layer. This results in a dense connectivity pattern where each neuron receives input from every neuron in the previous layer. Sparsely connected architectures can be beneficial because they can reduce the number of parameters in the model and improve computational efficiency, which can make training and inference faster and more resource-efficient. However, they can also be more difficult to train than fully connected architectures because of the reduced number of connections, and they may require specialized techniques to be implemented effectively.

In the context of convolutional neural networks, sparsely connected architectures can be achieved by using techniques such as dilated convolutions, depthwise separable convolutions, and group convolutions. These techniques allow for more efficient and sparse connectivity patterns within the convolutions, which can lead to improved performance and efficiency. In previous works, Transnet [5] and TransnetV2 [6] dilated convolutions have already been utilized. The main difference between standard convolutions and dilated convolutions, also known as atrous convolutions, is the spacing between the kernel elements. In standard convolutions, the kernel is applied to each input element in a sliding window fashion, with adjacent elements in the input grid being processed by adjacent elements in the kernel. In dilated convolutions, the kernel has gaps or holes between the elements, which allows the convolution to cover a larger input area with fewer kernel elements. This dilation factor can be adjusted, allowing dilated convolutions to have a larger receptive field than standard convolutions without increasing the number of parameters or sacrificing the resolution of the output. TransnetV2 [6] also employs 3D separable convolutions by stacking a 1 × 3 × 3 convolution in the spatial domain and a 3 × 1 × 1 convolution in the temporal domain. However, both models are supported by more than 4 million trainable parameters.

We propose the use of separable depthwise convolutions, such as those used in Mobilnet [1], as well as a residual self-attention block with the dual objective of substantially reducing the number of parameters without losing and, if possible, improving the performance of the original model. We use three NVIDIA RTX 3090 graphic cards as GPU support for training and inference. The computer has an i7 processor, with 96 GB RAM. Models are reduced by a factor between ×4 and ×5 the number of parameters, with a total training time of 6 h, using all IACC3 datasets and 30% split for test. Inference time is improved by a factor of 2.8, leading to infer 100 input frames in 0.8 s. To the best of our knowledge, this is the first contribution that proposes a parameter reduction without loss of performance in these models based on the aforementioned mechanisms.

## 4. Proposed Methods

### 4.1. Depthwise Separable Convolution

**Standard 3D convolution**. Similar to 2D convolution, 3D convolution applies a filter/kernel over the input data and calculates the dot product of the filter with each overlapping section of the input. The output of this operation is a new volume, where each element represents the dot product of the filter with the corresponding section of the input. In contrast to 2D convolution, where the input has two dimensions (width and height), 3D convolution operates on a 3D tensor with the dimensions depth, width, and height. The filter/kernel used in 3D convolution has a 3D shape and slides across the depth, width, and height dimensions of the input tensor. The output size of 3D convolution depends on the size of the input tensor, the size of the filter/kernel, and the stride used during the convolution operation.

Given a 3D feature matrix with shape (*l, w, h, c*), where *l, w, h* represents length, width, height and **c** denotes channels, the natural way of doing convolution operation on it would be using a filter with size *k* × *k* × *k*, where *k* is the side length of the filter, to go over the 3D matrix.

More formally, a standard convolution layer takes an input feature tensor **F** with shape ($l_{F},w_{F},h_{F},c_{F}$) and outputs a feature matrix **G** of size ($l_{G},w_{G},h_{G},c_{G}$). Notice that ($c_{F},c_{G}$) are the number of channels before and after the convolution. The number of parameters here should be of size *k* × *k* × *k* × $c_{F}$ × $c_{G}$, where *k* is the length side of the filter.

The output feature tensor for a standard 3D convolution is computed as:

(1) $$G_{x,y,z,n}=\sum_{i,j,k,m}K_{i,j,k,m,n}\cdot F_{x+i-1,y+j-1,z+k-1,m}$$

where *x, y, z* and *i, j, k* denote voxel’s spatial positions and *m* denotes the input channels and *n* the output channels.

The number of parameters of a standard 3D convolution (excluding bias) is:

(2) $$k\cdot k\cdot k\cdot c_{F}\cdot c_{G}$$

The computation cost would be:

(3) $$k\cdot k\cdot c_{F}\cdot c_{G}\cdot l_{F}\cdot w_{F}\cdot h_{F}$$

**3D Depthwise Convolution**. In 3D depthwise convolution, we decompose one 3D convolution operation into two steps: first, apply separate filters for each individual channel; second, stack the *c* feature maps obtained by the first step.

The output feature matrix for a 3D depthwise convolution is computed as:

(4) $$G_{x,y,z,m}^{{}^{\prime }}=\sum_{i,j,k,m}K_{i,j,k,m}^{{}^{\prime }}\cdot F_{x+i-1,y+j-1,z+k-1,m}$$

where *x, y, z* and *i, j, k* denote the spatial position of a voxel. K^′^ is a depthwise convolution kernel of size *k* × *k* × *k* × *c* (consisting of *c* filters). The *m*-th filter in K^′^ would be applied to the *m*-th channel in *F*. The output of the *m*-th filter becomes the *m*-th layer in G^′^.

The number of parameters of a standard 3D convolution (excluding bias) is:

(5) $$k\cdot k\cdot k\cdot c_{F}$$

The computation cost would be:

(6) $$k\cdot k\cdot k\cdot c_{F}\cdot l_{F}\cdot w_{F}\cdot h_{F}$$

**3D Pointwise Convolution** 3D pointwise convolution, also known as 1 × 1 × 1 convolution, is a type of convolutional operation that involves a filter/kernel with a size of 1 × 1 × 1 applied to the input tensor. In other words, 3D pointwise convolution operates on each individual voxel of the input tensor. Unlike regular 3D convolution, which uses a larger filter/kernel size to capture spatial information, pointwise 3D convolution is used to perform channel-wise operations on the input tensor. It is commonly used in deep learning models to increase or decrease the number of channels of a given tensor. It carries out a linear combination of layers of all depths. It is essentially fusing the split channels back together and activating the exchange of information adequately across channels. The number of parameters of a pointwise convolution is:

(7) $$c_{F}\cdot c_{G}$$

The computation cost would be:

(8) $$c_{F}\cdot c_{G}\cdot l_{F}\cdot w_{F}\cdot h_{F}$$

If we combine a depthwise and a pointwise convolution, the computational cost is:

(9) $$k\cdot k\cdot k\cdot c_{F}\cdot l_{F}\cdot w_{F}\cdot h_{F}+c_{F}\cdot c_{G}\cdot l_{F}\cdot w_{F}\cdot h_{F}$$

We then divide with the computational cost of a standard 3D convolution to obtain the reduction factor in computation terms:

(10) $$\frac{k\cdot k\cdot k\cdot c_{F}\cdot l_{F}\cdot w_{F}\cdot h_{F}+c_{F}\cdot c_{G}\cdot l_{F}\cdot w_{F}\cdot h_{F}}{k\cdot k\cdot k\cdot c_{F}\cdot c_{G}\cdot l_{F}\cdot w_{F}\cdot h_{F}}=\frac{k\cdot k\cdot k\cdot c_{F}+c_{F}\cdot c_{G}}{mk\cdot k\cdot k\cdot c_{F}\cdot c_{G}}=\frac{1}{c_{G}}+\frac{1}{k^{3}}$$

Assuming channel size $c_{G}$ = 3 and *k* = 3, the reduction is about 0.44. As soon as we increase the value of $c_{G}$, the term $\frac{1}{c_{G}}$ tends to zero, and the reduction tends to be 10 times the standard 3D convolution.

The effect of using a pointwise convolution after a depthwise convolution is that it helps to increase the expressiveness of the network while maintaining computational efficiency. The depthwise convolution captures spatial information from the input data, while the pointwise convolution combines this information and generates new features that can be used by the subsequent layers of the network. Using a pointwise convolution after a depthwise convolution helps to reduce overfitting in the network by reducing the number of parameters. This technique has been used in various successful neural network architectures, such as MobileNet [1] and ShuffleNet [22], to achieve high accuracy with low computational cost. The expressiveness of a network refers to its ability to represent a wide range of functions and approximate any input–output mapping to some degree of accuracy. In other words, the expressiveness of a network determines how well it can learn and represent complex patterns and relationships within the input data.

### 4.2. Residual Self-Attention

Self-attention is a mechanism in deep learning and natural language processing that helps the model to focus on different parts of the input when making predictions. The key, query, and value concepts are fundamental components of self-attention. In self-attention, the input is transformed into three vectors: key, query, and value. The key vector represents the importance of each sample in the input, the query vector is used to retrieve information from the key vector, and the value contains the actual information. During self-attention, the query tensor is used to compute the similarity scores between itself and each of the key tensors. The similarity scores are then used as weights to compute a weighted sum of the value vectors. The weighted sum is the final output of the self-attention mechanism. In essence, the key, query, value concept allows the self-attention mechanism to identify the most relevant parts of the input (represented by the key vector), retrieve information from those parts (using the query vector), and use that information to produce the final output (represented by the value vector).

The key, query, and value are denoted by *k(x)*, *q(x)*, and *v(x)* as follows:

(11) $$K_{ey}:k\left(x\right)=W_{k}x$$

(12) $$Q_{uery}:q\left(x\right)=W_{q}x$$

(13) $$V_{alue}:v\left(x\right)=W_{v}x$$

In our case, $x\in R^{NxTxHxWxC}$ is the tensor that represents the entry to the residual function. *N* is the batch dimension, *C* is the number of channels, *HxW* represents the spatial dimensions, and *T* the temporal dimension. We project *x* through the learnable query $W_{q}$, key $W_{k}$, and value $W_{v}$ layers. The number of filters of those layers defines the number of feature maps that will obtain. The attention map is a square matrix whose dimension will be equal to the number of features defined. If we define dimension “C” as the number of filters, the attention map will have dimension CxC, equivalent to a channel attention map. The self-attention map ($a_{ij}$) can be calculated as:

$$a_{ij}=\frac{\exp(k{\left(x_{i}\right)}^{T}q\left(x_{i}\right))}{\sum_{i=1}^{n}\exp(k{\left(x_{i}\right)}^{T}q\left(x_{i}\right)))}$$

where $a_{ij}$ indicates the correlative degree of attention between each *i* location and all other *j* locations. The output of the attention layer is $o_{j}$, where:

$$o_{j}=W_{u}\left(\sum_{i=1}^{N}a_{ij}v\left(x_{i}\right)\right)$$

$W_{u}$ represents a convolution utilized with the purpose of outputting a number of channels equal to that of the original input. The final output of the Residual Attention Block is represented by the formula:

y=x+o(x)

where *x* is the feature map input to the residual self-attention block and o(x) the output of the attention block.

### 4.3. Architectural Options

We use Transnet [5] and Transnetv2 [6] as base models, and we make modifications to both in different aspects of the architecture, with a double objective: (1) to reduce and optimize the number of model parameters as much as possible, and (2) to maintain or even improve the same performance as the original model, using as a metric the F1 score for Shot Boundary Detection.

We experiment with two major architectural modifications: (a) incorporating the use of depthwise separable convolutions and (b) adding a residual self-attention block, in which the feature map can be temporal, spatial, channel, or multiple. Within the depthwise option, we experiment with two versions: using a shared filter for all channels (v1) and using a different filter per channel (v2). In the residual self-attention option, we analyze two implementations, using 3D convolutions (v1) and using 1D convolutions (v2).

#### 4.3.1. Type of Depthwise Convolution

**Figure 1:**
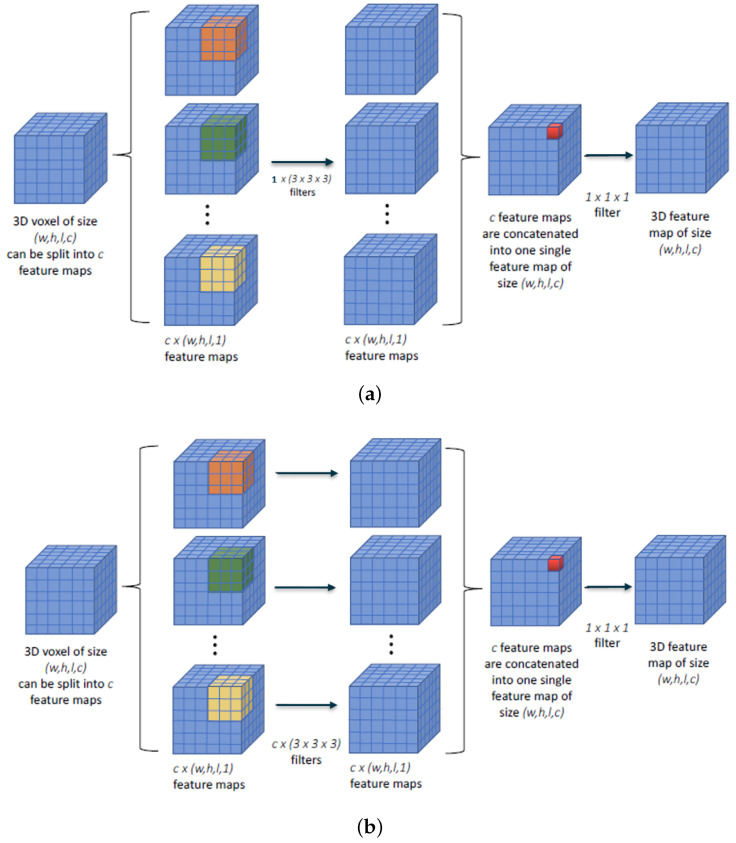
Depthwise separable convolution options. (**a**) Shared depthwise separable convolution. Only one filter is utilized and applied to all input channels; (**b**) common depthwise separable convolution. One different filter is applied to each input channel.

As in Figure 1a, it is the same filter for all channels. Without bias, the number of parameters if the input has three channels is 36 (27 of the 3D conv 3 × 3 × 3, plus 9 of the 1 × 1 × 1 pointwise convolution). With the option described in Figure 1b, where one filter is dedicated per input channel, we obtain 93 parameters (27 × 3 input channels, plus 9).

#### 4.3.2. Attention Blocks on a Different Axis

**Figure 2:**
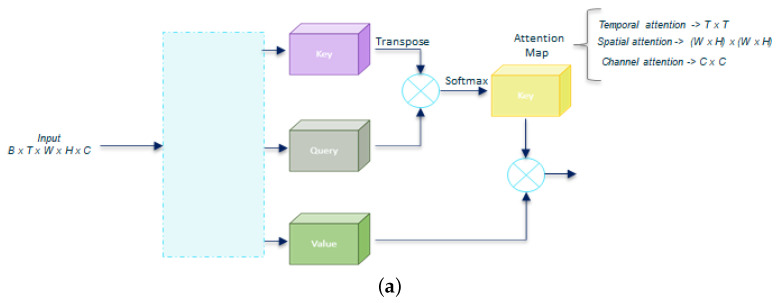

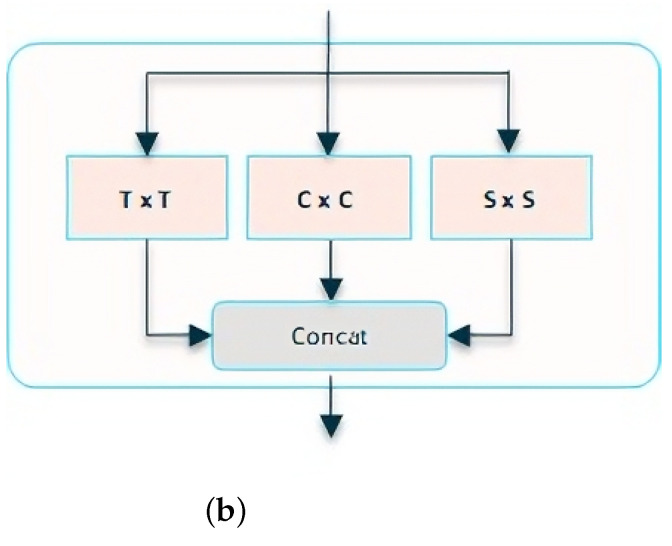
Residual self-attention types. (**a**) Same architecture but with attention maps dimensions depending on the axis selected: temporal, spatial, or channel; (**b**) multiple attention consists of applying several attention types and then concat in a single output.

We investigate the effect of the attention mechanism depending on the axis used to generate the attention map (see Figure 2a), distinguishing three options: temporal (T), spatial (S), and channel (C). We also tested a fourth option, named multiple, when we apply all three T, S, and C at once, as shown in Figure 2b.

#### 4.3.3. Residual Depthwise Dilated options

**Figure 3:**
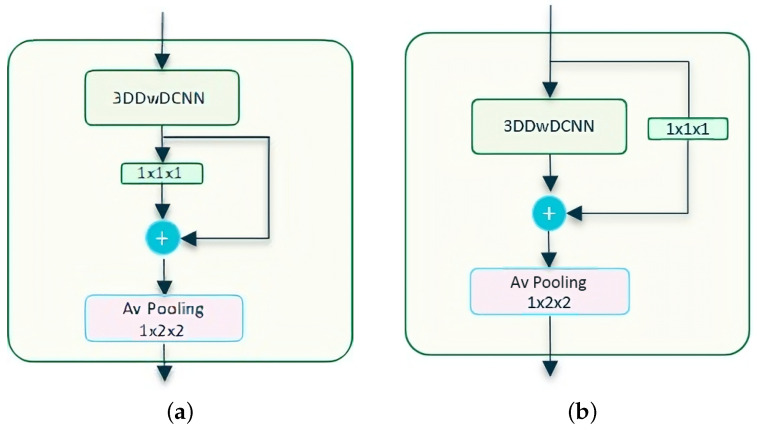
Component blocks for Depthwise Transnetv2 with self-attention. (**a**) Residual 3D Depthwise Dilated CNN block, **version A**. We add a residual connection around the pointwise convolution that follows the 3DDwDCNN layer; (**b**) Residual 3D Depthwise Dilated CNN block, **version B**. We add a residual connection around the 3DDwDCNN layer itself. We expect to mitigate the vanishing gradient, improve optimization, and allow the model to learn more complex features, all while using parameters more efficiently.

In the case of the Transnetv2 option, we tested with two options when applying residual connections: (a) residual connection on a pointwise after a Depthwise Dilated layer, Figure 3a, and option (b) residual connection on the Depthwise Dilated layer using a pointwise convolution to adjust the dimensions, Figure 3b.

#### 4.3.4. Residual Self-Attention Options

**Figure 4:**
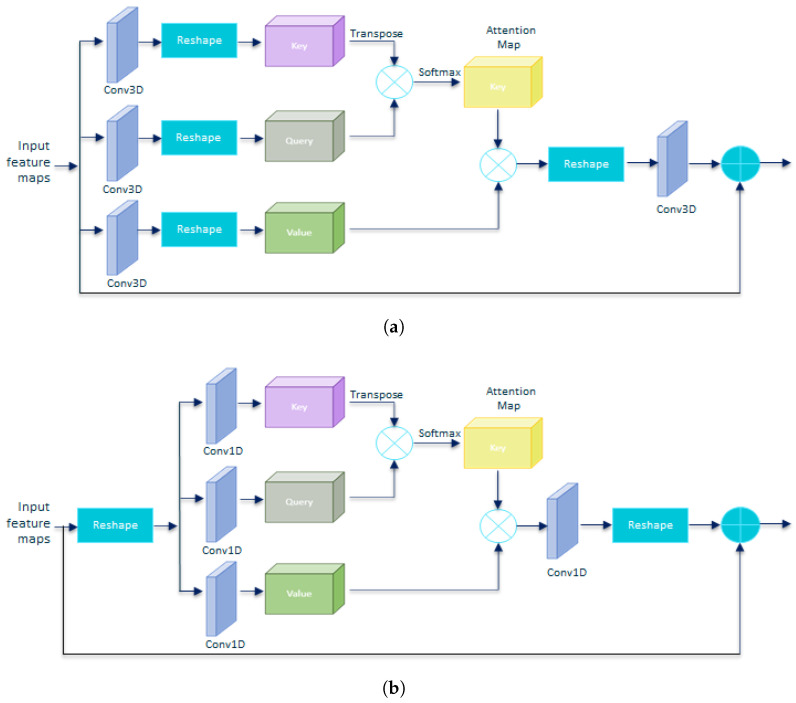
Residual self-attention options. (**a**) Residual attention block with 3D convolutions for key, query, and value tensors; (**b**) residual attention block with 1D convolutions for key, query, and value tensors.

We employ two residual attention block architecture options, depending on whether we use 3D (Figure 4a) or 1D (Figure 4b) convolutions for the key, query, and value tensors. Both architectures therefore differ in the number of trainable parameters as well as in the position of the architecture in which the tensors are reshaped.

### 4.4. Final Modified Networks

**Figure 5:**
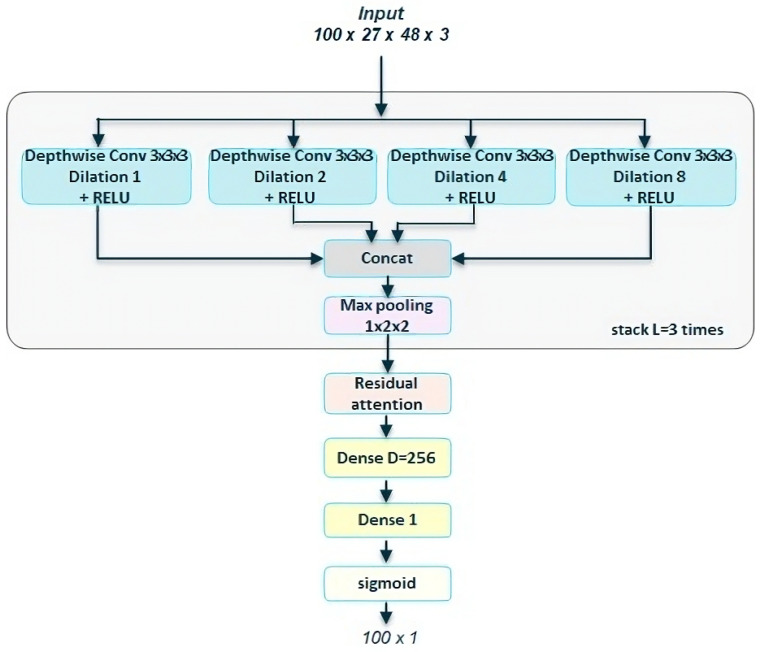
**Depthwise Transnet with self-attention.** Based on [5], we substitute the 3D dilated CNNs with 3D depthwise separable convolutions, where we set a filter per each input channel. We then add a residual self-attention block to the output of the Depthwise Dilated DCNN blocks.

**Figure 6:**
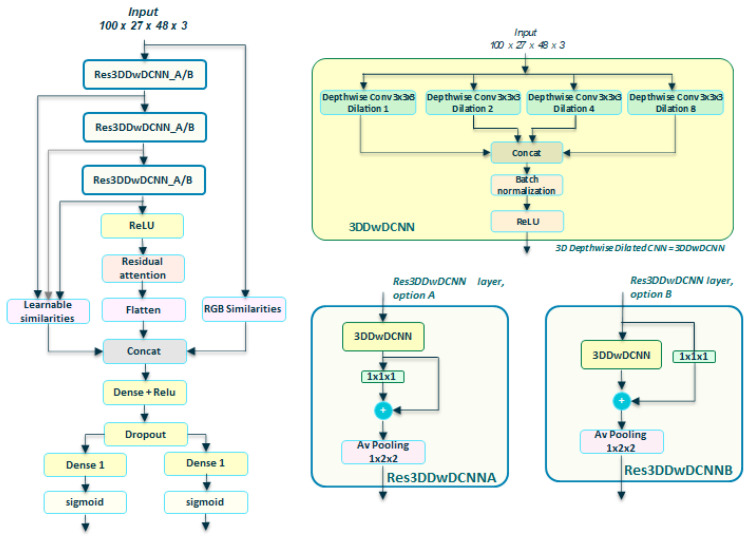
**DepthwiseTransnetv2 with self-attention.** Based on [6], we substitute the 3D dilated CNNs with 3D depthwise separable convolutions. We do not use stacked Dilated DCNN but Depthwise Dilated layers, with residual connections as in Figure 3a or Figure 3b. The Depthwise Dilated layers form part of a new layer, named Residual 3DDwCNN—Res3DDwDCNN—that is stacked L times, before the relu and residual self-attention block. We choose L = 3 as the best value.

We apply these changes to the Transnet and Transnetv2 networks. The nomenclature we use to name each version of the modified baseline when citing the experiments is the following:

- **Depthwisev{*****X*****}TransnetAttn{*****Type*****}v{*****Z*****}**, for our DepthwiseTransnet with attention (Figure 5)
- **Depthwisev{*****X*****}Transnetv2{*****Y*****}Attn{*****Type*****}v{*****Z*****}**, for our DepthwiseTransnetv2 with attention (Figure 6)
  where:
  – *X* refers to the depthwise option. *X* = 1 for one shared convolution for all channels option. *X* = 2 for the one different convolution for each channel option.
  – *Type* refers to the Attention type, with Type∈(T,S,C,M), and T: temporal, S: spatial (WxH), C: channel, M: multiple.
  – *Z* refers to attention convolutions. *Z* = 1 for Conv3D; *Z* = 2 for Conv1D
  – *Y*, with *Y* = A for ResDw3DCNN option A, and *Y* = A for ResDw3DCNN option B. This option only applies for Transnetv2.

Figure 5 shows our resulting Transnet-modified network. Note that we do not stack any dilated block as in the original. We just substitute the 3D dilated convolutions by our depthwise separable versions, then we concat the four, add a max pooling; then, the resulting block is stacked L times, with the best L equal to 3. After that stacks, we then apply the residual self-attention block. Then, we add two dense layers and a final sigmoid function that reduces the problem to a binary classification per each element of the resulting tensor.

Figure 6 shows our resulting Transnetv2-modified network. Note that we do not apply any Resnet-like residual connection over dilated blocks, as in the original. We just substitute the 3D dilated convolutions with our depthwise separable versions, resulting in a 3DDwDCNN layer. This layer is added as an average pooling after a residual connection in two possible options, A and B, that form the blocks ResDDwDCNNA and ResDDwDCNNB, respectively. The rest of the network keeps similar to the original, except we add a self-residual attention block after stacking three times the chosen Res3DDwDCNN block.

### 4.5. Networks Parameters

Table 1 shows the number of trainable parameters of the original models compared to the same models modified with just depthwise separable convolutions in the two versions analyzed. Table 2 shows the number of trainable parameters of the modified versions when using, besides depthwise separable convolutions, also a residual self-attention block of types temporal, spatial, channel, and multiple, differentiated by the version number of the attention block. Except when we use multiple attention, the rest of the combinations show a noticeable reduction in the number of parameters. In the experiments that we carried out in Section 5, we will have achieved our objectives if we match or exceed the F1 score results of the original models.

## 5. Experiments

The approach we follow is the same for all experiments. Given a dataset, we obtain the F1 score using the original Transnet [5] and Transnetv2 [6] models. We do not use data augmentation as the author does in [6]. We use the value we obtain as a reference with the original baseline model to later compare the performance with different architectural variations as indicated in Section 4.3.

All Depthwisev{*X*}TransnetAttn{*Type*}v{*Z*} variants are trained with an Adam optimizer and learning rate of 0.001, whereas Depthwisev{*X*}Transnetv2{*Y*}Attn{*Type*}v{*Z*} variants are trained with an SGD optimizer and learning rate of 0.01, except when Y = B. Then, we use Adam and a learning rate of 0.001. Regarding the experiments in Section 5.1 and Section 5.2, all of them were conducted using a split for validation and testing of the IACC3 dataset. On the other side, experiments in Section 5.3 were conducted using IACC3 [25] as a training dataset but tested with Clipshots [32], BBC [26], and RAI [33]. These experiments were conducted on a server equipped with three NVIDIA RTX 3090 as GPU support, as stated in the Motivation section.

### 5.1. Depthwisev{*X*}TransnetAttn{*Type*}v{*Z*} Experiments

**Figure 7:**
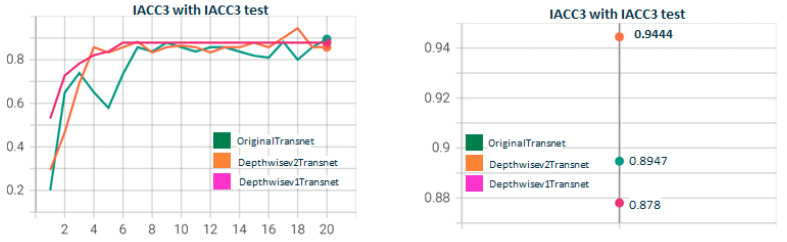
**Depthwisev1Transnet vs. Depthwisev2Transnet.** The F1 score obtained with the original version of Transnet is surpassed by only Depthwise version 2 (which uses a different filter per channel). When using version 1 (which only uses one filter for all channels) the F1 metric decreases. The version Depthwisev1 (shared filter) does not improve the original Transnet, but Depthwisev2 (shared filter) does. From the qualitative point of view, it is expected that if using Depthwise with a filter per channel, the model better captures the threshold patterns to differentiate short changes by visual similarity.

**Figure 8:**
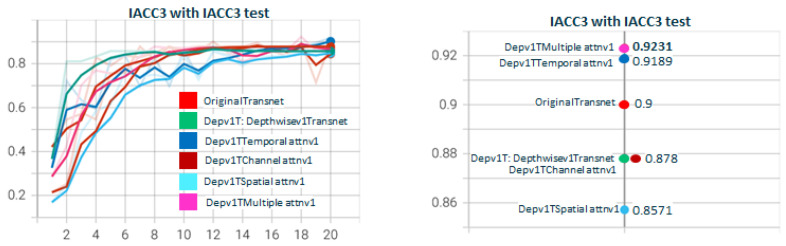
**Depthwisev1TransnetAttn*****Type*****v1 with Type∈(T,S,C,M) attention dimensions.** The graph shows how using several attention mechanisms affects the F1 score, using Depthwisev1Transnet. In this scenario, Depthwisev1 standalone does not improve the original Transnet, but when applying temporal and multiple attention, then it does. Notice that depthwisev1 and depthwisev1 with channel attention obtain the same score.

**Figure 9:**
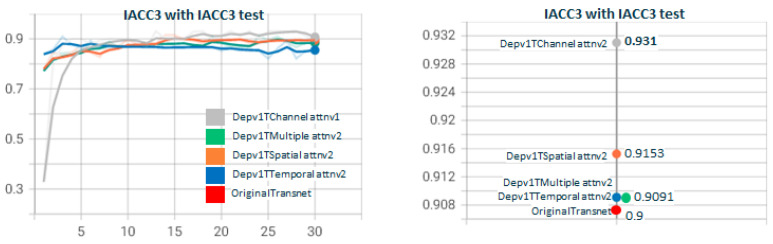
**Depthwisev1TransnetAttn*****Type*****v2 with Type∈(T,S,C,M) attention dimensions.** Depthwisev1Transnet with attention v2 for all types of attention. All attention types improve originalTransnet and Depthwisev1Transnet. Note attentionv2 improves attentionv1 attention with Depthwisev1Transnet.

**Figure 10:**
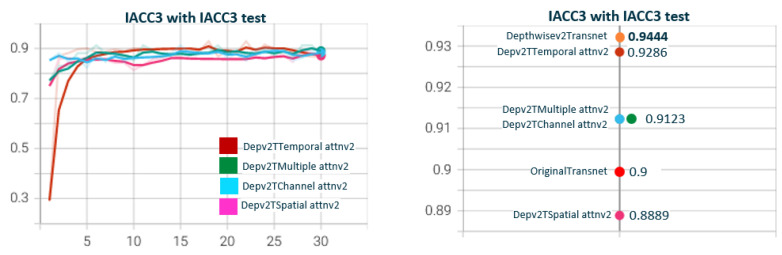
**Depthwisev2TransnetAttn*****Type*****v2 with Type∈(T,S,C,M) attention dimensions.** When using Depthwisev2Transnet with attention, no attention mechanism is able to improve Depthwisev2Transnet without attention.

In Figure 7, we show the results of the performance of DepthwiseTranset with the two depthwise versions. Depthwisev2Transnet (0.9444) improves the original transnet (0.8947). Depthwisev1Transnet gets close to the original but does not improve. We notice the high value obtained with Depthwisev2Transnet (0.9444) without attention. Depthwisev2Transnet really improved the original with a fraction of the parameters with no need for attention. In Figure 8, we test Depthwisev1Transnet with the attention block version1 (conv3D), comparing the performance of temporal, spatial, channel, and multiple attention. Depthwisev1Transnet, which did not improve the original, now improves with the multiple and temporal attention schemas. In Figure 9, we test Depthwisev1Transnet with the attention block version2 (conv1D). All attention types improve originalTransnet and Depthwisev1Transnet. Note that attentionv2 improves attentionv1 with Depthwisev1Transnet. We notice also that version 2 of attention surpasses the performance of v1. In Figure 10, we test Depthwisev2Transnet with the attention block version2 (conv1D). This time, as we suspected, due to the very high score obtained by Depthwisev2Transnet, none of the attention mechanisms allow us to improve the previously obtained result. In many cases, an attention block is able to improve the result when the model without attention solves a score below the original one. However, this premise does not always hold true. This leads us to think that under certain conditions, the attention block does not behave as expected.

### 5.2. Depthwisev{*X*}Transnetv2{*Y*}Attn{*Type*}v{*Z*} Experiments

**Figure 11:**
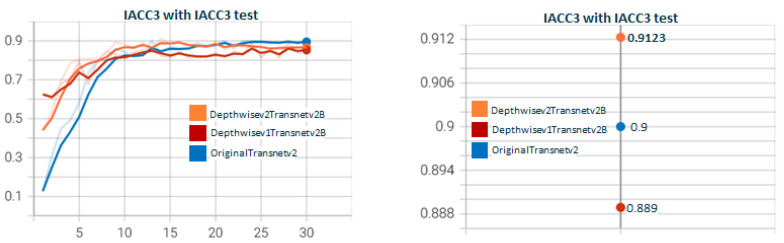
**Depthwisev1Transnetv2B vs. Depthwisev2Transnetv2B.** The graph compares DepthwiseTransnetv2B for Depthwise version v1 and v2. The F1 score obtained with the original version of transnetV2 is surpassed by Depthwise version 2. Depthwise version 2 (which uses a different filter per channel) is better than version 1 (which only uses one filter for all channels). TransnetV3 refers to the version with Res3DDwDCNN_B block. The version Depthwisev1 (shared filter) does not improve the original Transnetv2 but Depthwisev2 (shared filter) does. From the qualitative point of view, it is expected that if using Depthwise with a filter per channel, the model better captures the threshold patterns to differentiate short changes by visual similarity.

**Figure 12:**
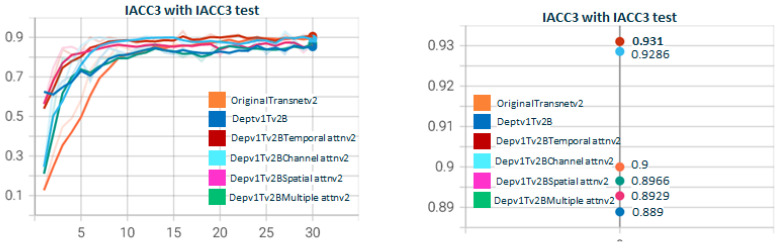
**Depthwisev1Transnetv2BAttn*****Type*****v2 with Type∈(T,S,C,M) attention dimensions.** In this graph, we show the comparison when using Depthwise option 1 (shared filter) and using the different self-attention dimensions in its version 2 (using 1D convolutions). It is observed that all attention mechanisms improve the metric obtained using Depthwisev1 without attention. The temporal self-attention and channel attention versions come to improve the original model. In this case, Depthwisev1 slightly worsens the original model, but with the use of attention (temporal or channel), it ends up improving it significantly. Note that Depthwisev2 with no attention (0.9123) is improved by Depthwisev1 with temporal (0.931) and spatial (0.9286) attention.

**Figure 13:**
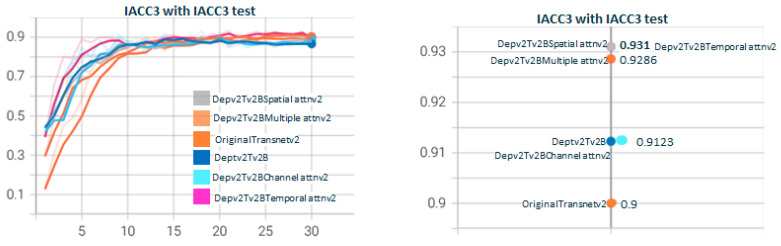
**Depthwisev2Transnetv2BAttn*****Type*****v2 with Type∈(T,S,C,M) attention dimensions.** In this graph, we show the comparison when using Dephtwisev2 (separate filter per input channel) and using the different self-attention options in its version 2 (using 1D convolutions). It is observed that not all attention mechanisms improve the metric obtained using Depthwisev2 without attention. Only temporal and spatial self-attention versions come to improve the original model up to 0.931. Note that we replicate value 0.931 using temporal attention, no matter the version of Depthwise.

**Figure 14:**
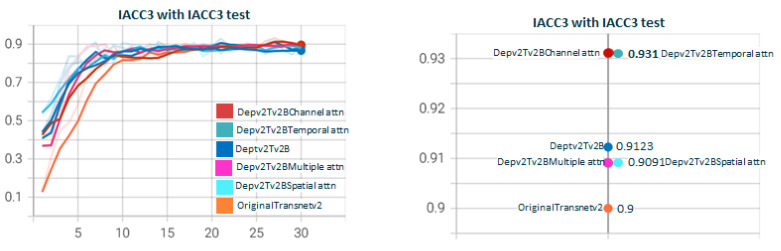
**Depthwisev2Transnetv2BAttn*****Type*****v1 with Type∈(T,S,C,M) attention dimensions.** In this graph, we show the comparison when using Depthwise option 2 (separate filter per input channel) and using the different self-attention options in its version 1 (using 3D convolutions). It is observed that temporal and channel dimensions for attention become predominant. Still, temporal attention continues as the one that obtains the maximum F1 score. Note that we replicate value 0.931 using temporal attention, no matter the version of Depthwise and no matter the version of attention (conv1d vs. conv3d).

**Figure 15:**
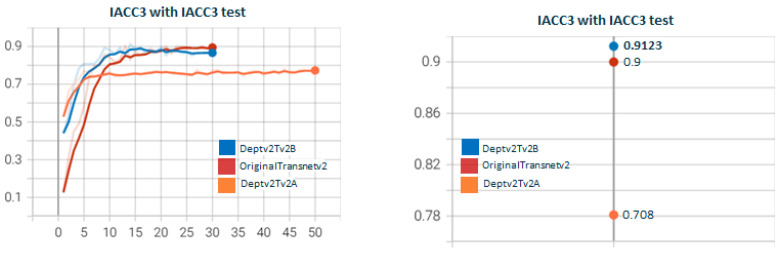
**Depthwisev2Transnetv2*****Y*** **with Y∈(A,B) ResDw3DDCNN layers.** In this graph, we show the comparison when using Depthwisev2 (separate filter per input channel) on TransnetV2 modified with blocks Res3DDwDCNN options A and B. In option A, the residual connection is conducted over a Pointwise conv at the output of the DepthwiseDilated layer; meanwhile, on option B, the residual connection is conducted over the DepthwiseDilated itself. The Depthwise layer captures features much better thanks to the concatenation of varying-size dilated convs, and therefore, adding the residual link helps to reduce overfitting and smooth gradient flow. This is the rationale behind the best result for option B versus A.

Figure 11 shows the test for DepthwiseTransnetv2B with the two Depthwise versions. As with Figure 7, Depthwisev2Transnetv2B (0.9213) improves the original transnet (0.9). Depthwisev2Transnetv2B gets close to the original but does not improve. In Figure 12, we test Depthwisev1Transnetv2B with attention block, comparing the performance of temporal, spatial, channel, and multiple attention. The version of the attention block is 2 (conv1D). Depthwisev1Transnetv2B, which did not improve the original, now improves with the channel and temporal attention schemas. In Figure 13, we test Depthwisev1Transnetv2B with attention block, comparing the performance of temporal, spatial, channel, and multiple attention. The version of the attention block is 2 (conv1D). All attention schemas still improve the Depthwisev1Transnetv2B performance, which in turn already improved the original. In Figure 14, we test the same as in Figure 13 but with the attention block version 1 based on Conv3D. The performance gets similar to the ones with v2. In Figure 15, we test comparing the effect of using ResDw3dDDCNN version A versus ResDw3dDDCNN version B. Version B of the Residual Depthwise block clearly improves version A.

### 5.3. Other Experiments

We experiment using the dataset IACC3 for training, and ClipshotsTests, BBC, and RAI for tests. The results are shown in Table 3, Table 4, and Table 5, respectively.

When we tested with ClipShotsTest, Table 3, the Depthwisev2 modification was very similar to the original but did not improve it. If we analyze the results carefully, the results are still similar, but they are not improved either. ClipShotsTest is a dataset with a high number of false negatives, that is, non-annotated transitions. This is the reason for the poor results even with the original model. The network cannot learn where a transition is to the extent that we tell it one thing and the opposite at the same time with the same classification. Obviously, adding an attention mechanism does not correct the basic error of using a badly annotated dataset.

When we tested with BBC, Table 4, a much better-annotated dataset than ClipShots, we noticed that the F1 score of the Transnet model (0.931) is higher than that of TransnetV2 (0.916). These two values are the ones we use from the start in the comparison with our modifications. Experimentally, we verified that the multiple attention version (0.944) improves Transnet and that the Depthwisev2 version (0.927) does the same in the case of TrasnetV2.

When we test with RAI, the result is similar to that obtained by BBC. RAI is a dataset that also has a high number of unannotated transitions, although not to the extent of ClipShots. In this case, Transnet (0.897) is improved by the Depthwise version with channel attention (0.911) and TransnetV2 (0.905) with spatial attention (0.915). That is, the original results are improved with a fraction ×4 of the total parameters.

**Figure 16:**
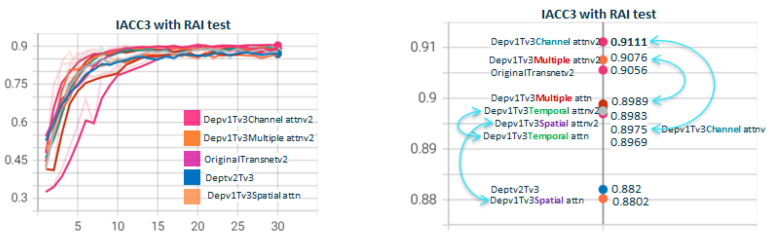
**Depthwisev1Transnetv2BAttn{*****Type*****}v{*****Z*****} with Type∈(T,S,C,M) and Z∈(1,2).** For the same attention dimension, version 2 of the attention block, based on 1D convolutions, performs slightly better than version 1, based on 3D convolution. Being the input to the self-attention block, a 5-rank tensor, the use of conv1D requires reshaping the input to a 3-rank tensor: batch dimension, intermediate dimension, and channel dimension. The channel dimension acquires the value of the dimension that is used to generate the feature map, be it T, S, or C. The intermediate one will contain the product of the rest. In the case of using conv3d, the input tensor is not formatted, but the outputs to K, Q, and V are formatted. The loss of resolution in the projection of 3D convolutions makes attention map performance higher using conv1D relative to conv3d.

In addition, and in order to test variants 1 and 2 of the attention blocks, in Figure 16, we compare again the attention blocks v1 and v2 for the different attention schemas but using IACC3 for training and RAI for the test. This time, version 2 of the attention blocks always improves the corresponding schema in version 1.

## 6. Conclusions

In this work, we present two new models for video shot boundary detection. Those are based on modified versions of Transnet [5] and Transnetv2 [6], with the use of depthwise separable convolution and visual self-attention. Our objective is to significantly reduce the number of parameters used in the original models and improve the inference time of the models. This is necessary for segmenting live streaming content, so that the segments are generated with a minimum delay with respect to the live signal, and thus, can be used in use cases such as viewing the content from the beginning (start-over). In order to improve the possible loss of performance that we could find, we incorporate a residual attention block, with different variants, which we call temporal, spatial, channel, and multiple, depending on the dimension of the input tensor to the block on which the attention map is conformed.

We have investigated two variants of the depthwise separable convolution. Version 1 employs only one filter for all input channels, while version two employs a different filter per channel. In general, version 2 is better than version 1 because it is not forced to extract features from a single shared filter for all channels. In the case of TransnetV2, one of the modifications we made is to incorporate a block called Res3DDwDCNN, which we investigated in two versions, A and B, differentiated in how a residual connection is constituted. We observed that option B is better than A because the residual connection is made directly on the 3DDwDCNN layer, using a pointwise convolution for dimension adjustment.

In the case of the attention block, we investigate two versions, v1, and v2. Version v1 uses 3D convolutions, while v2 uses 1D convolutions. This leads to having to reform the tensors at different points in the block. We observe that version v2 works slightly better than v1 because with the use of pointwise convolutions, no reduction of the input tensor is performed, and therefore, the attention map is generated without losing representative capacity, already very reduced due to the use of depthwise convolutions in the previous stage.

All experiments have been performed using the IACC3 [25] dataset for training. For testing, we have used the IACC3 split, as well as ClipShotsTests [32], BBC [26], and RAI [33].

When we tested with the IACC3 split, the use of the Depthwise version 2 modification without attention block improved the F1 metric obtained with the original model. The attention block even improved the metric obtained using Depthwise version 1 to be similar to the one obtained using Depthwise version 2 with attention. We also verified this when testing with BBC for the case of Depthwisev2Transnetv2B.

In the case of testing with RAI and ClipShots, the metrics obtained with the Depthwise versions are similar to the original ones. It should be noted that the Depthwise versions are therefore matching and even improving the F1 metrics of the original models to a fraction between ×4 and ×5 the number of parameters of the original models.

With respect to the impact on the use of the attention block, in all cases, the F1 metric of the original model is improved, except with Depthwisev2Transnet. The DepthwiseTransnetv2 score was so high that none of the other types of care were able to improve it. Besides this, we noticed that when there is an improvement, it is not always using the same type of attention. These are relevant facts that we leave for further research and we think are correlated. Possible improvements to address this issue in the attention block would be to use a parametrizable dimension attention map, $L=2^{x}$, with x∈(7,8,9,10), so L∈(128,256,512,1024). In reshaping the output tensor of the key and query layers, we place on axis 1 the product of all dimensions and on axis 2, the L value that optimizes the F1 metric. The attention map would be obtained by scaling the real one, of size LxL, to the temporal one, of size TxT. Another possible improvement would be to use a pointwise residual connection multiplied by a trainable α scalar value, adjusted to provide the best F1 metric by eliminating the rigidity of a direct residual connection.

Also relevant is the disparity of F1 results obtained depending on the test dataset used. This fact is also evident in the original papers. We believe that for this task to be tackled with better guarantees, it would be convenient to develop a single standardized benchmark dataset for both training and testing that would serve as a reference in a similar way as other datasets, such as Imagenet, in the image classification task. For all tests, we use three NVIDIA RTX 3090 graphic cards as GPU support for training and inference. The computer has an i7 processor, with 96GB RAM. Models are reduced by a factor between ×4 and ×5 the number of parameters, with a total training time of 6 h, using all IACC3 datasets and 30% split for test. Inference time is 0.8 s for an input of 100 frames.

## Figures and Tables

**Table 1:** Parameters with depthwise options.

		Depthwise		
	Original	v1	v2	
Transnet	4.614.593	895.665	902.385	
TransnetV2	4.763.970	1.060.526	1.067.246	ResDw3DCNN_A
		1.031.042	1.037.762	ResDw3DCNN_B

**Table 2:** Parameters with Self-Attention options.

		Depthwisev{X}							
		X = 1				X = 2			
		Attn{Type}v2				Attn{Type}v2			
		T	C	S	M	T	C	S	M
	Transnet:	936.065	1.043.889	897.033	2.855.129	942.785	1.050.609	916.455	2.861.849
TransnetV2:	ResDw3DDCNN_A	1.100.926	1.208.750	1.061.894	3.019.990	1.107.646	1.215.470	1.068.614	3.026.710
	ResDw3DDCNN_B	1.1071.422	1.179.266	1.032.410	2.990.506	1.078.162	1.185.986	1.038.130	2.997.226

**Table 3:** Best F1 score using IACC3 for training and ClipShotsTest for the test.

Dataset/Annotations	Original	Depthwisev2	+Tempv2	+Spav2	+Chav2	+Mulv2
Transnet	**0.661**	0.650	0.625	0.627	0.656	0.649
TransnetV2	**0.694**	0.659	0.686	0.686	0.687	0.648

**Table 4:** Best F1 score using IACC3 for training and BBC for test.

Dataset/Annotations	Original	Depthwisev2	+Tempv2	+Spav2	+Chav2	+Mulv2
Transnet	**0.931**	0.921	0.930	0.912	0.925	**0.944**
TransnetV2	0.916	**0.927**	0.920	0.916	0.922	0.913

**Table 5:** Best F1 score using IACC3 for training and RAI for test.

Dataset/Annotations	Original	Depthwisev2	+Tempv2	+Spav2	+Chav2	+Mulv2
Transnet	**0.897**	0.894	0.911	0.896	0.894	**0.910**
TransnetV2	0.905	0.903	0.905	0.897	**0.915**	0.905

## Data Availability

Datasets and code are available under request to corresponding authors.
